# Porcine Collagen Matrices for the Treatment of Multiple Gingival Recessions: A Case Report With 4 Years Follow‐Up

**DOI:** 10.1155/crid/9967849

**Published:** 2026-04-16

**Authors:** Mauricio Andrés Tinajero Aroni, Suzane Cristina Pigossi, Fausto Mauricio Tinajero Camacho, Marcelo Villacis Valencia, Joni Augusto Cirelli, Thamiris Cirelli

**Affiliations:** ^1^ International University of Ecuador (UIDE), Quito, Pichincha, Ecuador; ^2^ Department of Periodontology and Implantodontology, School of Dentistry, Federal University of Uberlandia (UFU), Uberlândia, MG, Brazil, ufu.br; ^3^ Hemisferios University, Quito, Pichincha, Ecuador; ^4^ Department of Diagnosis and Surgery, School of Dentistry at Araraquara, Sao Paulo State University (UNESP), Araraquara, SP, Brazil, unesp.br; ^5^ Department of Dentistry, University Center of Associated School (UNIFAE), São João da Boa Vista, SP, Brazil

**Keywords:** collagen, gingival recession, graft, gum

## Abstract

**Background:**

This case report aimed to evaluate the clinical performance of Mucoderm and Fibro‐Gide collagen matrices (CMs) as alternatives to connective tissue graft (CTG) in the treatment of multiple gingival recessions (GRs).

**Methods:**

A 37‐year‐old man, systemically healthy and a nonsmoker patient, with multiple bilateral GR (recession type 1 class [RT1] or Miller Class I) involving teeth 12–16 and 22–26 was surgically treated. Envelope type of coronally advanced flap (CAF) was used in teeth 14–16 and 23–26 associated with Fibro‐Gide matrix on the right side and Mucoderm matrix on the left side.

**Results:**

Complete root coverage was observed in all GR after 60 days of postoperative, except for tooth 22. In the Fibro‐Gide matrix‐treated side, a greater increase in keratinized tissue (KT) volume was visually observed after 60 days. However, an apical displacement of approximately 0.50 mm of the marginal gingiva was observed in teeth 14, 15, 24–26 with 12 months, showing stability of the results after 4 years of follow‐up. Moreover, no differences regarding KT volume and gingival color/texture were visually observed between both matrices after 5 years.

**Conclusion:**

The Mucoderm and Fibro‐Gide CMs demonstrated adequate clinical performance for GR coverage in this case report.

## 1. Introduction

Gingival morphology is critical and contributes significantly to the final dental and facial esthetics. In this context, the presence of gingival recessions (GRs), with consequent root exposure and morphological changes in periodontal tissues, can result in esthetic sequelae, high patient frustration, presence of caries and noncarious cervical lesions [1, 2]. Studies reported that 88% of patients aged 65 and over 50% of patients aged 18–64 have one or more sites with GR, and their presence and extension increase with age [3].

Periodontal plastic surgery techniques are commonly employed to address GR by increasing gingival tissue dimensions and achieving predictable root coverage in clinical practice [2]. However, among these techniques, the conventional coronally advanced flap (CCAF) combined with a connective tissue graft (CTG) is considered the gold standard technique offering superior root coverage and keratinized tissue (KT) gain [4]. However, the wound at the palatal donor site for harvesting the CTG often leads to increased patient discomfort, postsurgical complications, mainly bleeding from the donor site and prolonged surgical time. In addition, the amount of available tissue in the donor area may be limited, decreasing the number of sites that can be treated in a single surgical procedure [5].

Based on these CTG disadvantages, new biomaterials are being developed to substitute for the CTG and minimize patient morbidity. Collagen matrices (CMs) have been proposed as autogenous graft substitutes to avoid a second surgical area, ensure unlimited availability, decrease surgical time, and reduce postoperative complications due to the lack of a donor site [6, 7]. All CMs are decellularized, deproteinized and avascular, but differ regarding their resorption process [8]. Mucoderm (Botiss Biomaterials, Berlin, Germany) is a novel tridimensional porcine‐derived acellular dermal matrix, composed of natural types I and III collagen with a natural collagen structure that resembles the human connective tissue and offers a safe alternative to CTG in a diverse range of soft tissue grafting indications [9]. After implantation, Mucoderm is continuously remodeled into the patient’s soft tissue [10]. Similarly, the Geistlich Fibro‐Gide (Geistlich Pharma AG, Wolhusen, Switzerland), a porcine, porous, resorbable and volume‐stable CM, was specifically designed for soft‐tissue regeneration. According to the manufacturer, this CM is made of reconstituted collagen and undergoes smart chemically cross‐linking to improve its volume stability while maintaining good biocompatibility. Previous studies have demonstrated that both biomaterial exhibits predictable tissue integration, low inflammatory response, high biocompatibility, and no reports of severe adverse reactions [9–12]. Therefore, both biomaterials offer viable alternatives to CTG, with potential advantages in terms of handling, reduced morbidity, and soft‐tissue regeneration outcomes.

This case report aimed to evaluate the clinical performance of Mucoderm and Fibro‐Gide CMs as alternatives to CTG in the treatment of multiple GRs.

## 2. Case Report

**Figure 1:**
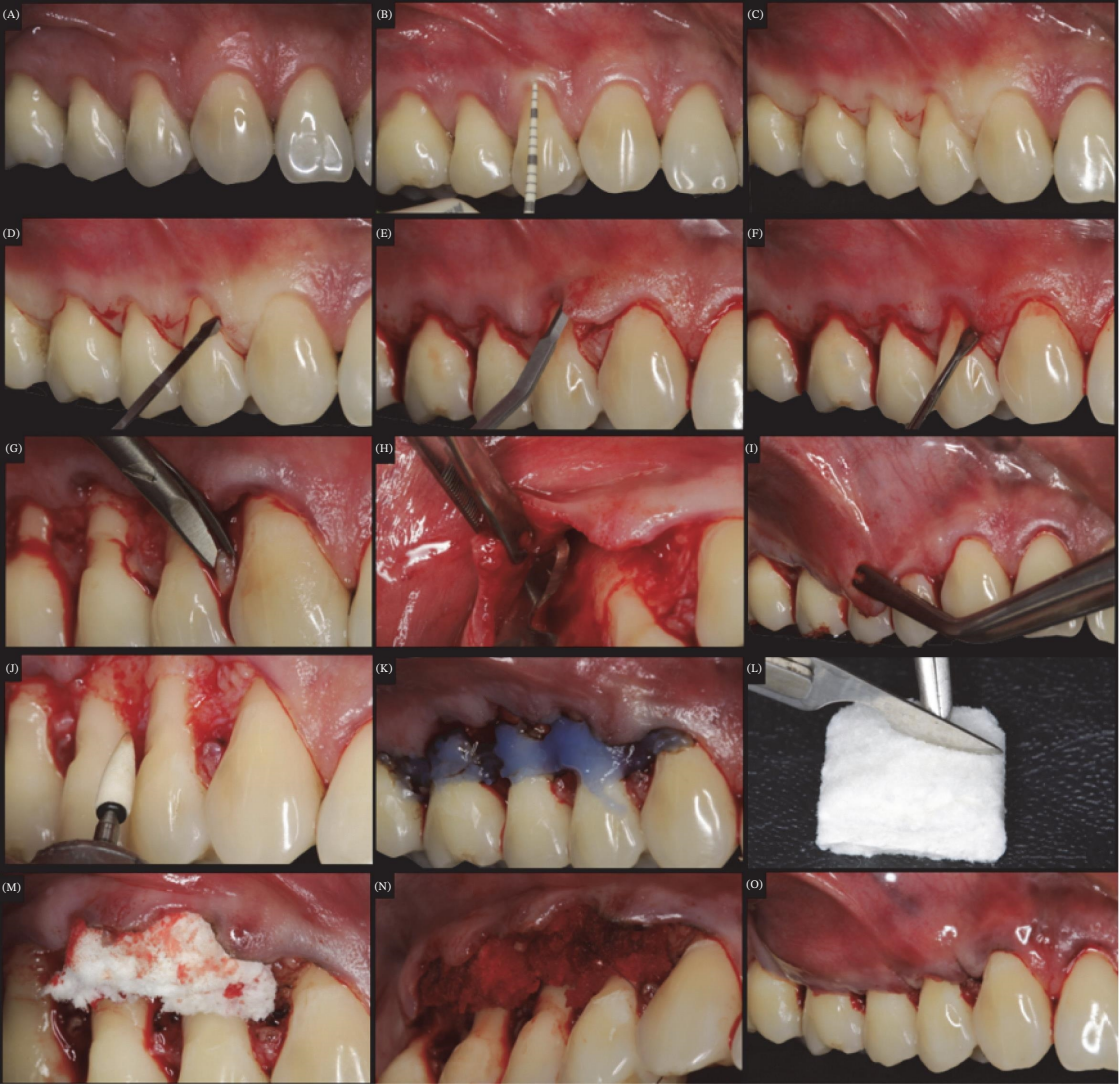
Surgical sequence for the treatment of the right side gingival recessions with Fibro‐Gide. (A) Initial condition, presence of recessions in teeth 12, 14, 15, and 16. (B) Recession size measurement with a periodontal probe. (C) V‐shaped incisions. (D) Sulcular incision with Micro‐blades (Vyper). (E) Flap detachment with micro‐blades (SM69). (F) Mucoperiosteal detachment with tunelizers up to the height of the mucogingival junction. (G) Papillary de‐epithelialization with delicate tissue scissors. (H) Partial flap release. (I) Stress‐free flap. (J) Smoothness of the non‐carious cervical lesion. (K) Dentin biomodification with 37% phosphoric acid. (L) Fibro‐Gide membrane trimming. (M) Fibro‐Gide positioning over the main recessions. (N) Fibro‐Gide moisturized with blood, after suturing. (O) final aspect, after suture and coronal repositioning of the flap.

**Figure 2:**
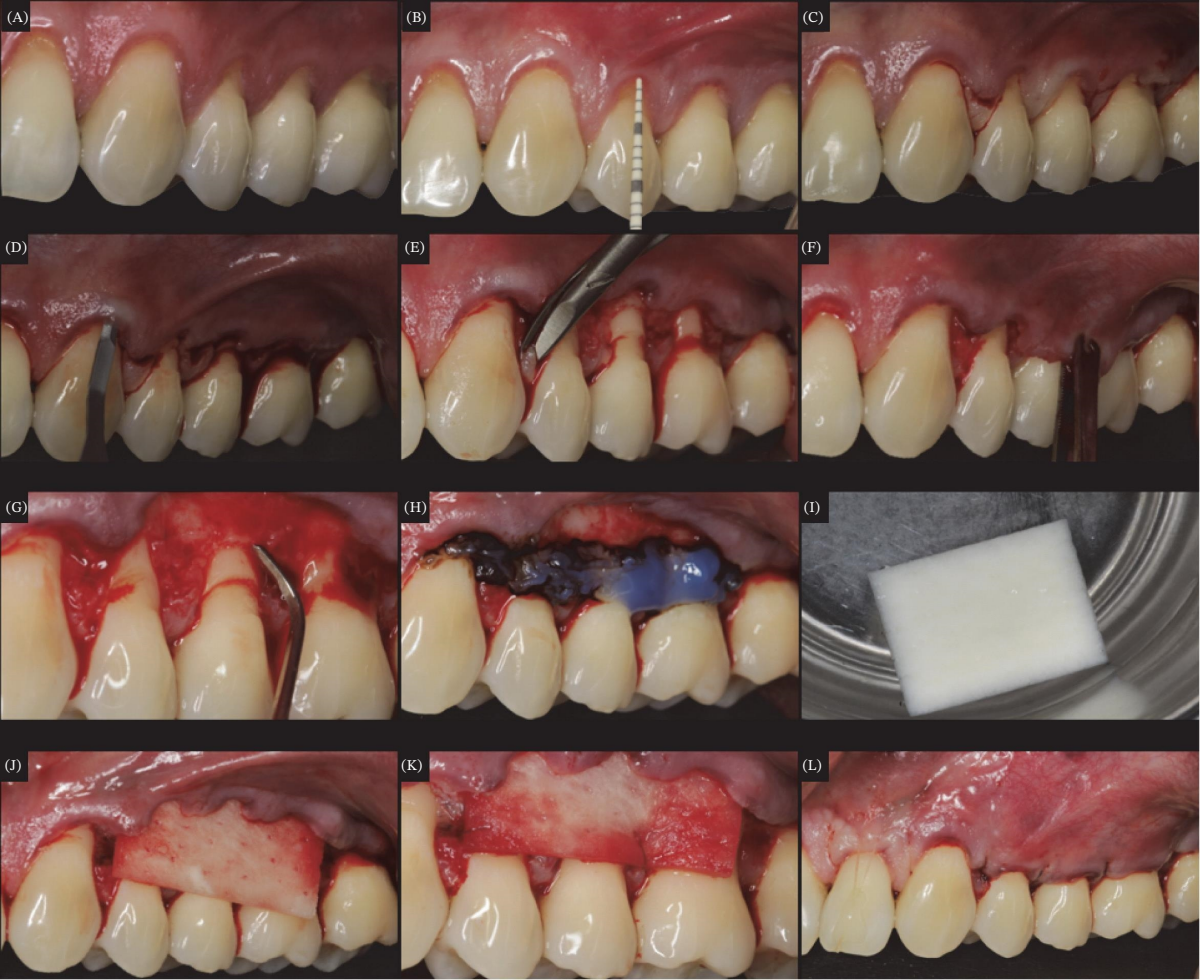
Surgical sequence for the treatment of the left side gingival recessions with Mucoderm. (A) Initial condition, presence of recessions in teeth 22, 24, 25, and 26. (B) Recession size measurement with a periodontal probe. (C) Drawing of the V‐shaped incisions, imitating the anatomy of the papillae. (D) Sulcular incisions with microblades (Vyper). (E) De‐epithelialization of the papillae using delicate tissue scissors. (F) Evidence of flap release, without tension. (G) Root surface planning using manual curettes. (H) Dentin biomodification with 37% phosphoric acid. (I) Hydration of Mucoderm in saline for 20 min. (J) Mucoderm positioned on the main recessions. (K) Mucoderm after suture. (L) Final aspect, after suture and coronal repositioning of the flap.

This case report was conducted in accordance with the Declaration of Helsinki, following the CARE guidelines and informed consent was obtained from the participant included in the study. A 37‐year‐old man, systemically healthy and a nonsmoker patient, sought treatment at the Periodontal Clinic in the School of Dentistry at Araraquara, Sao Paulo State University – UNESP, complaining of dental esthetics in the anterior region of the maxilla. The clinical examination revealed that the patient had multiple bilateral GR (recession type 1 class [RT1] (Table 1) [13] or Miller Class I 14]) in teeth 12, 14–16, 22−26 (Figures 1A and 2A). Considering that the patient presented with an adequate width of KT at baseline (>2 mm), and aiming to reduce postoperative morbidity, the treatment proposed consisted of a surgical approach of the GRs with an envelope type of CAF associated with CMs. After receiving instructions regarding two commercial matrices available for his case from different companies (Fibro‐Gide and Mucoderm), the patient was informed that the biomaterial used in the procedure was of animal (porcine) origin and consented to use both materials, one on each side of the maxilla. The sides were randomly selected using an online tool (www.sealedenvelope.com), being Fibro‐Gide used on the right side and Mucoderm on the left side. Before the surgical procedure, the patient was submitted to a preliminary periodontal evaluation, scaling and root planning, as well as for instructions on oral hygiene. At baseline (T0), probing depth was ≤3 mm at all sites, with no bleeding on probing.

**Table 1:** Gingival recession depth.

Periods	Fibro‐Gide				Mucoderm				
	16	15	14	12	22	23	24	25	26
Baseline	4 mm	3 mm	3,5 mm	1 mm	2 mm	1 mm	2 mm	3 mm	3 mm
4 years	0 mm	0.5 mm	0.5 mm	0 mm	1 mm	0 mm	0.5 mm	0.5 mm	0.5 mm

Clinical measurements were recorded at the mid‐buccal point of the affected teeth (Figures 1B,E and 2B) at baseline (prior to surgery), 2 months, 12 months, and 4 years after the procedure. All measurements were performed by the same investigator using the same type of periodontal probe, which was blinded to the biomaterials used on each side. (UNC 15, Hu‐Friedy, Chicago, IL, USA). The following parameters were evaluated [15]: (1) GR depth (GRD); (2) pocket probing depth (PPD); (3) clinical attachment level (CAL). PPD and CAL were analyzed at the distobuccal, mid‐buccal and mesiobuccal aspects of surgical sites.

The primary outcome of this case report was the stability of root coverage over time, assessed through reduction and maintenance of GRD. The secondary outcomes included: changes in CAL; PPD; clinical observation of KT width and thickness appearance; gingival color and texture integration; long‐term stability of the gingival margin over 4 years. KT changes were clinically and visually assessed during follow‐up appointments.

About 1h before surgery, a prophylactic dose of antibiotic (Amoxicillin, 2 g) associated with a steroidal anti‐inflammatory agent (Dexamethasone, 4 mg) was administered. Intra and extraoral asepsis were achieved using 0.12% and 2% chlorhexidine gluconate, respectively. Local anesthesia was induced using a 4% articaine solution with epinephrine 1:100.000 (Nova DFL). For envelope type of CAF, the initial horizontal incision in “V” shape (reproducing the papillae shape) was made into the adjacent interdental papillae at the CEJ of the tooth with GR using a stainless fine steel micro blade Swann‐Morton model (SM69) (Figures 1C,D and 2C) [16]. A partial‐thickness flap was created on both sides by sharp dissection, including teeth 13–16 and 23–26. The GRs of teeth 12 and 22 were not included.

The mesiodistal length of the incision was extended to provide easy access to the denuded root once vertical incisions were not used (Figures 1E,F and 2D). The adjacent papillae were de‐epithelized (Figures 1G and 2E) and the flap was extended apically to the mucogingival junction (MGJ), allowing a tension‐free coronal flap position (Figures 1H,I and 2F). The root surface debridement was made using Gracey curettes (Hu‐Friedy, RJ, Brazil), especially in the areas previously exposed to the oral environment (Figure 2G). The flattening of noncarious cervical lesions was done with a FF 2135 (FG ‐ KG Sorensen) drill (Figure 1J), and 37% phosphoric acid (Dentsply) (Figure 1K) was used for root surface treatment (Figures 1K and 2H).

After the flap preparation, the Mucograft (Figure 1L) and Fibro‐gide matrices were cut and trimmed to the exact size of both side defects, such that ≥1 mm of the surrounding tissue was covered. Fibro‐Gide matrix was used on the right side and the Mucoderm matrix on the left side. The Mucoderm matrix was hydrated in saline for 20 min, following the manufacturer’s instructions (Figure 2I). Then, both CM were positioned over the main recessions (premolar teeth) (Figures 1M and 2J) and immobilized in the surgical site with a subperiosteal suture (Marlin violet, absorbable, DSM 11, 6‐0) (Figures 1N and 2K).

For teeth 12 and 22, a modified coronally advanced tunnel (MCAT) technique [17, 18] was used without CM. Intrasulcular incisions were performed, and mucoperiosteal flaps were raised using sharp tunnel elevators, preserving the papillae (Quinelato, Brazil). The flap was extended beyond the MGJ and under each papilla, allowing a tension‐free flap mobilization in the coronal direction.

A double‐loop sling suture (Resorba Sutures Resolon, blue, USP 6/0) was made in the flap interdental region, positioning the flap coronal to the CEJ (16–26 teeth) (Figures 1O and 2L). The same operator performed the surgeries on both sides. Patients were prescribed 0.12% chlorhexidine gluconate (Periogard, Colgate, Brazil) and instructed to rinse gently twice daily for 15 days. Tooth brushing was discontinued in the surgical area during this time. An antibiotic (Amoxicillin, 500 mg, 8/8 h) was prescribed for 7 days to prevent possible postoperative infection. A nonsteroidal anti‐inflammatory (Nimesulide, 100 mg, 12/12 h) and an analgesic (Dypirone, 500 mg, 6/6 h) were also prescribed. After 15 days, the postsurgery course was uneventful, and the sutures were removed.

**Figure 3:**
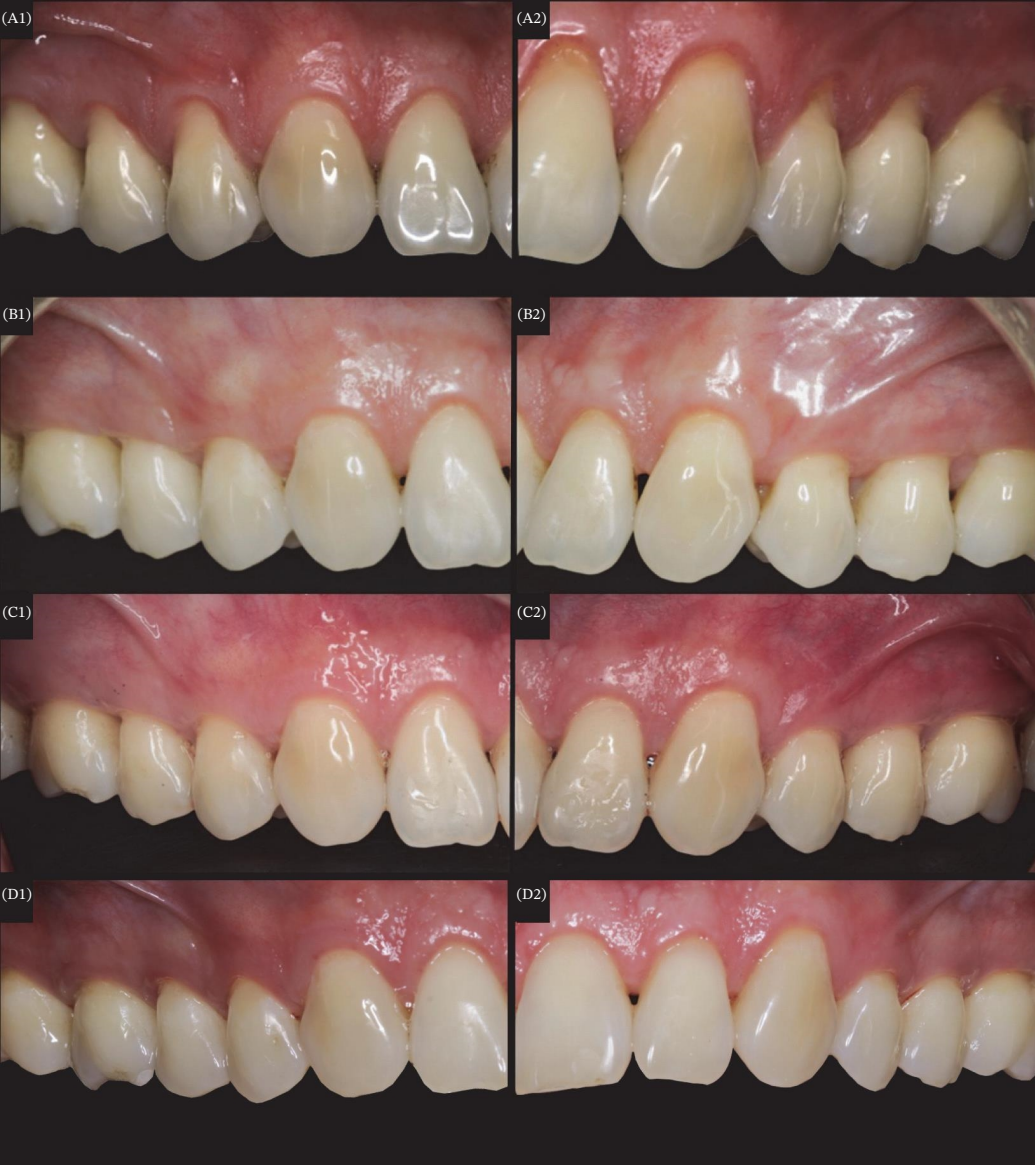
Comparison of the clinical aspect between Fibro‐Gide (A2–D2) and Mucoderm (A1–D1), at the baseline (T0—A), 2 months (T1—B), 12months (T2—C) and after 4 years (T3—D) of follow‐up.

In comparison with the initial aspect (Figure 3A), complete root coverage was observed in all GR after 60 days of postoperative, except for tooth 22 (GRD = 1 mm). In the Fibro‐Gide matrix side, a greater increase in KT volume was visually observed after 60 days (Figure 3B). However, an apical displacement of 0.50 mm of the marginal gingiva was observed in teeth 14, 15, 24–26 after 12 months, showing stability after 4 years of follow‐up (Table 1). Moreover, no differences regarding KT volume and gingival color/texture were visually observed between both matrices after 12 months (Figure 3C). Finally, the patient was extremely satisfied with the clinical results obtained on both sides after 4 years (Figure 3D).

## 3. Discussion

As an alternative to CTG, xenogeneic soft tissue substitutes, such as porcine‐derived 3D collagen‐based matrices, have been proposed due to their unlimited availability, decreased surgical time, lack of a donor site, and reduced postoperative complications [19]. Based on these advantages, the present case report compared the use of two porcine CMs (Fibro‐Gide and Mucoderm) as an alternative to CTG in the treatment of multiple GRs. Although CTG remains the gold standard, the patient in this case presented with adequate width of KT at baseline, making the use of biomaterials a clinically acceptable option. This favorable baseline situation supported the decision to select CMs to minimize postoperative morbidity while still achieving predictable root coverage outcomes [20].

According to the results, both CMs demonstrated adequate clinical performance for the GR coverage, with no differences regarding KT volume and gingival color/texture after 12 months. Moreover, as described in other studies in the literature, the use of both matrices ensured color and texture matching, allowed treatment of multiple sites in one surgical intervention, and prevented the need for a donor site, reducing operatory time [21, 22].

The CM effectiveness has been evaluated in controlled clinical studies comparing the treatment of Miller Class I and II (RT1) single recessions with CAF alone or combined with CM [23, 24]. Moreira et al. [24] reported a GRD of 0.75 mm ±0.76 for CM (baseline 3.16 ± 0.65) and 0.89 ± 0.52 for CAF alone (baseline 3.14 ± 0.51) after 6 months. Stefanini et al. (2016) [23] also obtained a GRD of 0.83 ± 0.99 for CM alone (baseline 3.46 ± 0.90) in comparison to 0.93 ± 1.10 for CCAF alone (baseline 3.34 ± 1.00). On the other hand, better clinical results were observed by Cardaropoli et al. [25] with a smaller final GRD of 0.23 mm ±0.47 for CM (baseline 3.09 ± 0.63) and 0.09 mm ±0.20 for CTG (baseline 3.05 ± 0.65) after 12 months. Similar results were observed in this case report using Fibro‐Gide and Mucoderm matrices once complete root coverage was obtained in all GR that received a CM, after 60 days of postoperative. However, an apical displacement of the marginal gingiva of 0.50 mm was observed in most of the teeth after 12 months. Clinical trials studies that made a comparison between xenogeneic matrices and CTG demonstrating after 1 year of follow‐up decreased of mean root coverage of recession at the test sites when comparing the outcomes at 6 and 12 months [26, 27]. This finding may be explained by the phenomena known as “creeping attachment,” where usually CTG demonstrates a tendency of gradual KT width increase over time, producing also higher mean root coverage of recession [27]. It is important to emphasize that the present case report did not include a CTG control group; therefore, no direct comparison with CTG can be established, and the observed outcomes should be interpreted within the descriptive nature of this report.

Collagenous matrices vary concerning composition, three‐dimensional structure, elasticity, and mechanical stability. The collapse of a CM after implantation may impair host cell migration and blood vessel penetration, negatively influencing tissue degradation and integration, as well as extracellular matrix production in the interior of the biomaterial [28]. Then, volume stability and interconnectivity of pores are considered important requirements for biomaterials used in tissue augmentation [29, 30]. In this context, a novel volume‐stable CM with rapid tissue integration (Geistlich Fibro‐Gide) was developed to increase the width of soft connective tissue [28]. However, to our knowledge, no clinical study has yet been published evaluating the GR coverage using the Fibro‐Gide matrices. In three preclinical studies, the Fibro‐Gide showed a gain of the thickness of newly formed tissues adjacent to the bone and dental implants after 3 months, but not so in the long‐term, that is, at 6 months. Similarly, in this case report, the Fibro‐Gide matrix side visually showed a greater KT volume increase than the Mucoderm matrix after 60 days. However, no quantitative thickness or volumetric measurements were performed, and no differences regarding KT volume were visually observed between both matrices after 12 months.

Mucoderm, a tridimensional matrix composed of natural types I and III collagen without any artificial cross‐linking, was developed and showed significant integration with surrounding tissues and excellent revascularization properties in vitro [31, 32]. A study in dogs evaluated the effectiveness of Mucoderm in soft tissue thickening compared to CTG and showed that the CM was statistically similar to the CTG in terms of soft tissue volume and thickness increase. Furthermore, the 3D tissue measurements revealed that the difference in volume increase was non‐significant between CTG (11.36 ± 9.26 mm) and Mucoderm (8.67 ± 13.67 mm) groups after 10 months of soft tissue thickening [33]. In the same way, a prospective pilot study showed that the use of Mucoderm during implant surgery ensured KT width augmentation of 7.86 ± 3.22 mm after 30 days. Moreover, no membrane exposures or wound healing complications were observed during the postoperative phase and, after 1 year, the mean KT width augmentation obtained was preserved (5.67 ± 2.12 mm) [9].

The results of the present case report are similar to the literature, since both matrices ensured adequate root coverage and KT gain after 12 months, suggesting that Mucoderm and Fibro‐Gide can be used as alternatives to CTG. However, it is noteworthy that additional studies are needed to estimate the clinical potentiality and describe the long‐term limits of the technique, strengthening the scientific evidence of both matrices. In addition, it is important to note that there are some disadvantages regarding the use of exogenous materials that include additional costs to the patient and the occurrence of graft contraction during the healing phase [22]. Moreover, the Mucoderm matrix needs to be hydrated in saline for 20 min, following the manufacturer’s instructions [34], which increases the operatory time.

The envelope type of flap was chosen in this case report to avoid vertical incisions that may prejudice the blood supply of the overlying tissue [35]. This type of flap ensures the surgical margin stability critical to the success of the root coverage procedures [16]. Furthermore, after healing, the vertical releasing incisions often result in an unesthetic appearance with a scar, which can be even more unsatisfactory for the patient than the root exposure itself [16]. Zucchelli et al. [36] showed in a controlled randomized clinical trial that the envelope type of CAF was associated with an increased probability of achieving complete root coverage and a better postoperative course. Likewise, in teeth 12 and 22, the MCAT technique was made to avoid vertical releasing incisions and papillary incisions, aiming to improve vascularization of the area. This technique ensures a premature esthetic result and adequate healing; however, it should be avoided in large recessions to prevent excessive graft exposure [17, 18].

Mechanical root surface modification was made in this case report to smooth irregularities and grooves of the root surface and minimize cementum toxicity [37]. In combination with the mechanical treatment, 37% phosphoric acid was carefully applied to the root surface to remove the smear layer produced by root instrumentation, expose collagen fibrils of the dentin matrix, facilitating the formation of new connective tissue attachment [38, 39], and remove cytopathic substances that inhibit human gingival fibroblast growth from infected cementum [40]. Moreover, preoperative medications were administered in this case report to modulate edema and prevent infection [41]. Markiewicz et al. [42], concluded, in a meta‐analysis, that perioperative administration of corticosteroids produces a mild to moderate reduction in edema and trismus than controls during the early and late postoperative period after third molar surgery.

## 4. Conclusion

In this case report, the Fibro‐Gide and Mucoderm matrices demonstrated adequate clinical performance and long‐term stability in the treatment of multiple GRs. These findings suggest that porcine CMs may represent viable biomaterial options for root coverage procedures; however, further clinical trials are needed to confirm the outcomes of this case report.

## Author Contributions

**Mauricio Andrés Tinajero Aroni, Suzane Cristina Pigossi, Fausto Mauricio Tinajero Camacho, Marcelo Villacis Valencia, Joni Augusto Cirelli, and Thamiris Cirelli**: material preparation, data collection and analysis. **Mauricio Andrés Tinajero Aroni and Suzane Cristina Pigossi**: original draft. All authors commented on previous versions of the manuscript and contributed to the study conception and design.

## Funding

The authors declare that no funds, grants, or other support were received during the preparation of this manuscript.

## Disclosure

All authors read and approved the final manuscript.

## Ethics Statement

This study was performed in line with the principles of the Declaration of Helsinki. Informed consent was obtained from the participant included in the study. The authors affirm that the human research participant provided informed consent for publication of the images.

## Conflicts of Interest

The authors declare no conflicts of interest.

## Data Availability

The data that support the findings of this study are available upon request from the corresponding author. The data are not publicly available due to privacy or ethical restrictions.

## References

[1] Allen E. P. and Miller P. D.Jr, Coronal Positioning of Existing Gingiva: Short Term Results in the Treatment of Shallow Marginal Tissue Recession, Journal of Periodontology. (1989) 60, no. 6, 316–319, 10.1902/jop.1989.60.6.316, 2-s2.0-0024674959. 2778599

[2] Tonetti M. S. and Jepsen S. , Working Group 2 of the European Workshop on P. Clinical Efficacy of Periodontal Plastic Surgery Procedures: Consensus Report of Group 2 of the 10th European Workshop on Periodontology, Journal of Clinical Periodontology. (2014) 41, no. no. 15, S36–S43. 24640999 10.1111/jcpe.12219

[3] Kassab M. M. and Cohen R. E. , The Etiology and Prevalence of Gingival Recession, Journal of the American Dental Association. (2003) 134, no. 2, 220–225, 10.14219/jada.archive.2003.0137, 2-s2.0-0037945621. 12636127

[4] Cairo F. , Pagliaro U. , and Buti J. , et al.Root Coverage Procedures Improve Patient Aesthetics. A Systematic Review and Bayesian Network Meta-Analysis, Journal of Clinical Periodontology. (2016) 43, no. 11, 965–975, 10.1111/jcpe.12603, 2-s2.0-84991253781. 27454460

[5] Chambrone L. , Chambrone D. , Pustiglioni F. E. , Chambrone L. A. , and Lima L. A. , Can Subepithelial Connective Tissue Grafts be Considered the Gold Standard Procedure in the Treatment of Miller Class I and II Recession-Type Defects?, Journal of Dentistry. (2008) 36, no. 9, 659–671, 10.1016/j.jdent.2008.05.007, 2-s2.0-48549103599. 18584934

[6] Huang J. P. , Liu J. M. , Wu Y. M. , Chen L. L. , and Ding P. H. , Efficacy of Xenogeneic Collagen Matrix in the Treatment of Gingival Recessions: A Systematic Review and Meta-Analysis, Oral Diseases. (2019) 25, no. 4, 996–1008, 10.1111/odi.12949, 2-s2.0-85052801142. 30076680

[7] Tinajero-Aroni M. A. , Pigossi S. C. , Pimentel-Oliveira G. J. , Pichotano E. C. , and Chierici-Marcantonio R. A. , Association Between Platelet-Rich Fibrin and Collagen Matrix for Root Coverage: Case Series, International Journal of Interdisciplinary Dentistry. (2021) 14, no. 1, 48–51, 10.4067/S2452-55882021000100048.

[8] Rothamel D. , Schwarz F. , and Fienitz T. , et al.Biocompatibility and Biodegradation of a Native Porcine Pericardium Membrane: Results of In Vitro and In Vivo Examinations, The International Journal of Oral & Maxillofacial Implants. (2012) 27, no. 1, 146–154. 22299091

[9] Papi P. and Pompa G. , The Use of a Novel Porcine Derived Acellular Dermal Matrix (Mucoderm) in Peri-Implant Soft Tissue Augmentation: Preliminary Results of a Prospective Pilot Cohort Study, BioMed Research International. (2018) 2018, 1–9, 10.1155/2018/6406051, 2-s2.0-85049743709. PMC6077540 30112412

[10] Rothamel D. , Benner M. , and Fienitz T. , et al.Biodegradation Pattern and Tissue Integration of Native and Cross-Linked Porcine Collagen Soft Tissue Augmentation Matrices - An Experimental Study in the Rat, Head & Face Medicine. (2014) 10, no. 1, 10.1186/1746-160X-10-10, 2-s2.0-84898927371. PMC3984020 24670219

[11] Barbeck M. , Lorenz J. , and Kubesch A. , et al.Porcine Dermis-Derived Collagen Membranes Induce Implantation Bed Vascularization Via Multinucleated Giant Cells: A Physiological Reaction?, Journal of Oral Implantology. (2015) 41, no. 6, e238–e251, 10.1563/aaid-joi-D-14-00274, 2-s2.0-84949939453. 25546240

[12] Bienz S. P. , Vaquette C. , and Ioannidis A. , et al.Tissue Integration and Biodegradation of Soft Tissue Substitutes with and without Compression: An Experimental Study in the Rat, Clinical Oral Investigations. (2023) 27, no. 1, 313–328, 10.1007/s00784-022-04726-0. 36255492 PMC9877052

[13] Jepsen S. , Caton J. G. , and Albandar J. M. , et al.Periodontal Manifestations of Systemic Diseases and Developmental and Acquired Conditions: Consensus Report of workgroup 3 of the 2017 World Workshop on the Classification of Periodontal and Peri-Implant Diseases and Conditions, Journal of Clinical Periodontology. (2018) 45, no. no. 20, S219–S229. 29926500 10.1111/jcpe.12951

[14] Miller P. D.Jr, A Classification of Marginal Tissue Recession, The International Journal of Periodontics & Restorative Dentistry. (1985) 5, no. 2, 8–13. 3858267

[15] Aroca S. , Molnar B. , and Windisch P. , et al.Treatment of Multiple Adjacent Miller Class I and II Gingival Recessions With a Modified Coronally Advanced Tunnel (MCAT) Technique and a Collagen Matrix or Palatal Connective Tissue Graft: A Randomized, Controlled Clinical Trial, Journal of Clinical Periodontology. (2013) 40, no. 7, 713–720, 10.1111/jcpe.12112, 2-s2.0-84878555730. 23627374

[16] Zucchelli G. and De Sanctis M. , Treatment of Multiple Recession-Type Defects in Patients with Esthetic Demands, Journal of Periodontology. (2000) 71, no. 9, 1506–1514, 10.1902/jop.2000.71.9.1506, 2-s2.0-0034276745. 11022782

[17] Raetzke P. B. , Covering Localized Areas of Root Exposure Employing the ”Envelope“ Technique, Journal of Periodontology. (1985) 56, no. 7, 397–402, 10.1902/jop.1985.56.7.397, 2-s2.0-0022099253. 3894614

[18] Tozum T. F. and Dini F. M. , Treatment of Adjacent Gingival Recessions With Subepithelial Connective Tissue Grafts and the Modified Tunnel Technique, Quintessence International. (2003) 34, no. 1, 7–13. 12674352

[19] Lima R. S. , Peruzzo D. C. , Napimoga M. H. , Saba-Chujfi E. , Dos Santos-Pereira S. A. , and Martinez E. F. , Evaluation of the Biological Behavior of Mucograft(R) in Human Gingival Fibroblasts: An In Vitro Study, Brazilian Dental Journal. (2015) 26, no. 6, 602–606, 10.1590/0103-6440201300238, 2-s2.0-84954051235. 26963203

[20] Halim F. C. and Sulijaya B. , Allogenic Acellular Dermal Matrix and Xenogeneic Dermal Matrix as Connective Tissue Graft Substitutes for Long-Term Stability Gingival Recession Therapy: A Systematic Review and Meta-Analysis, European Journal of Dentistry. (2024) 18, no. 2, 430–440, 10.1055/s-0043-1772778. 37848072 PMC11132762

[21] Zucchelli G. , Clauser C. , De Sanctis M. , and Calandriello M. , Mucogingival Versus Guided Tissue Regeneration Procedures in the Treatment of Deep Recession Type Defects, Journal of Periodontology. (1998) 69, no. 2, 138–145, 10.1902/jop.1998.69.2.138, 2-s2.0-0031993011. 9526912

[22] Atieh M. A. , Alsabeeha N. , Tawse-Smith A. , and Payne A. G. , Xenogeneic Collagen Matrix for Periodontal Plastic Surgery Procedures: A Systematic Review and Meta-Analysis, Journal of Periodontal Research. (2016) 51, no. 4, 438–452, 10.1111/jre.12333, 2-s2.0-85027922670. 26547393

[23] Stefanini M. , Jepsen K. , and de Sanctis M. , et al.Patient-Reported Outcomes and Aesthetic Evaluation of Root Coverage Procedures: A 12-Month Follow-up of a Randomized Controlled Clinical Trial, Journal of Clinical Periodontology. (2016) 43, no. 12, 1132–1141, 10.1111/jcpe.12626, 2-s2.0-84995755423. 27717210

[24] Moreira A. R. O. , Santamaria M. P. , and Silverio K. G. , et al.Coronally Advanced Flap With or Without Porcine Collagen Matrix for Root Coverage: A Randomized Clinical Trial, Clinical Oral Investigations. (2016) 20, no. 9, 2539–2549, 10.1007/s00784-016-1757-8, 2-s2.0-84975701662. 26917493

[25] Cardaropoli D. , Tamagnone L. , Roffredo A. , and Gaveglio L. , Treatment of Gingival Recession Defects Using Coronally Advanced Flap With a Porcine Collagen Matrix Compared to Coronally Advanced Flap With Connective Tissue Graft: A Randomized Controlled Clinical Trial, Journal of Periodontology. (2012) 83, 321–328, 10.1902/jop.2011.110215, 2-s2.0-84857848495. 21721988

[26] Rakasevic D. L. , Milinkovic I. Z. , Jankovic S. M. , Soldatovic I. A. , Aleksic Z. M. , and Nikolic-Jakoba N. S. , The Use of Collagen Porcine Dermal Matrix and Connective Tissue Graft With Modified Coronally Advanced Tunnel Technique in the Treatment of Multiple Adjacent Type I Gingival Recessions: A Randomized, Controlled Clinical Trial, Journal of Esthetic and Restorative Dentistry. (2020) 32, no. 7, 681–690, 10.1111/jerd.12624. 32706184

[27] Vincent-Bugnas S. , Borie G. , and Charbit Y. , Treatment of Multiple Maxillary Adjacent Class I and II Gingival Recessions With Modified Coronally Advanced Tunnel and a New Xenogeneic Acellular Dermal Matrix, Journal of Esthetic and Restorative Dentistry. (2018) 30, no. 2, 89–95, 10.1111/jerd.12337, 2-s2.0-85029427839. 28901687

[28] Caballe-Serrano J. , Zhang S. , Ferrantino L. , Simion M. , Chappuis V. , and Bosshardt D. D. , Tissue Response to a Porous Collagen Matrix Used for Soft Tissue Augmentation, Materials. (2019) 12, no. 22, 10.3390/ma12223721. PMC6888327 31718004

[29] Ashworth J. C. , Mehr M. , Buxton P. G. , Best S. M. , and Cameron R. E. , Cell Invasion in Collagen Scaffold Architectures Characterized by Percolation Theory, Advanced Healthcare Materials. (2015) 4, no. 9, 1317–1321, 10.1002/adhm.201500197, 2-s2.0-84932198581. 25881025 PMC4529738

[30] Ashworth J. C. , Mehr M. , Buxton P. G. , Best S. M. , and Cameron R. E. , Parameterizing the Transport Pathways for Cell Invasion in Complex Scaffold Architectures, Tissue Engineering Part C: Methods. (2016) 22, no. 5, 409–417, 10.1089/ten.tec.2015.0483, 2-s2.0-84969190253. 26888449 PMC4870607

[31] Pabst A. M. , Lehmann K. M. , Walter C. , Kruger M. , Stratul S. I. , and Kasaj A. , Influence of Porcine-Derived Collagen Matrix on Endothelial Progenitor Cells: An In Vitro Study, Odontology. (2016) 104, no. 1, 19–26, 10.1007/s10266-014-0186-x, 2-s2.0-84953359033. 25487653

[32] Park J. S. , Pabst A. M. , Ackermann M. , Moergel M. , Jung J. , and Kasaj A. , Biofunctionalization of Porcine-Derived Collagen Matrix Using Enamel Matrix Derivative and Platelet-Rich Fibrin: Influence on Mature Endothelial Cell Characteristics In Vitro, Clinical Oral Investigations. (2018) 22, no. 2, 909–917, 10.1007/s00784-017-2170-7, 2-s2.0-85022209731. 28695450

[33] Schmitt C. M. , Matta R. E. , and Moest T. , et al.Soft Tissue Volume Alterations after Connective Tissue Grafting at Teeth: The Subepithelial Autologous Connective Tissue Graft versus a Porcine Collagen Matrix - A Pre-Clinical Volumetric Analysis, Journal of Clinical Periodontology. (2016) 43, no. 7, 609–617, 10.1111/jcpe.12547, 2-s2.0-84971377859. 26990041

[34] Kasaj A. , Levin L. , and Stratul S. I. , et al.The Influence of Various Rehydration Protocols on Biomechanical Properties of Different Acellular Tissue Matrices, Clinical Oral Investigations. (2016) 20, no. 6, 1303–1315, 10.1007/s00784-015-1614-1, 2-s2.0-84944556383. 26434650

[35] Bruno J. F. , Connective Tissue Graft Technique Assuring Wide Root Coverage, The International Journal of Periodontics & Restorative Dentistry. (1994) 14, no. 2, 126–137. 7928129

[36] Zucchelli G. , Mele M. , Mazzotti C. , Marzadori M. , Montebugnoli L. , and De Sanctis M. , Coronally Advanced Flap With and Without Vertical Releasing Incisions for the Treatment of Multiple Gingival Recessions: A Comparative Controlled Randomized Clinical Trial, Journal of Periodontology. (2009) 80, no. 7, 1083–1094, 10.1902/jop.2009.090041, 2-s2.0-68049139054. 19563288

[37] Pini-Prato G. , Baldi C. , and Pagliaro U. , et al.Coronally Advanced Flap Procedure for Root Coverage. Treatment of Root Surface: Root Planing Versus Polishing, Journal of Periodontology. (1999) 70, no. 9, 1064–1076, 10.1902/jop.1999.70.9.1064, 2-s2.0-0033193126. 10505810

[38] Selvig K. A. , Ririe C. M. , Nilveus R. , and Egelberg J. , Fine Structure of New Connective Tissue Attachment Following Acid Treatment of Experimental Furcation Pockets in Dogs, Journal of Periodontal Research. (1981) 16, no. 1, 123–129, 10.1111/j.1600-0765.1981.tb00956.x, 2-s2.0-0019515563. 6453967

[39] Polson A. M. and Proye M. P. , Effect of Root Surface Alterations on Periodontal Healing. II. Citric Acid Treatment of the Denuded Root, Journal of Clinical Periodontology. (1982) 9, no. 6, 441–454, 10.1111/j.1600-051X.1982.tb02105.x, 2-s2.0-0020212453. 6960020

[40] Olson R. H. , Adams D. F. , and Layman D. L. , Inhibitory Effect of Periodontally Diseased Root Extracts on the Growth of Human Gingival Fibroblasts, Journal of Periodontology. (1985) 56, no. 10, 592–596, 10.1902/jop.1985.56.10.592, 2-s2.0-0022134178. 3903101

[41] Queiroz T. P. , Santos P. L. D. , and Esteves J. C. , et al.Dipirona versus Paracetamol No Controle da dor pós-Operatória, Revista de Odontologia da UNESP. (2013) 42, no. 2, 78–82, 10.1590/S1807-25772013000200002.

[42] Markiewicz M. R. , Brady M. F. , Ding E. L. , and Dodson T. B. , Corticosteroids Reduce Postoperative Morbidity After Third Molar Surgery: A Systematic Review and Meta-Analysis, Journal of Oral and Maxillofacial Surgery. (2008) 66, no. 9, 1881–1894, 10.1016/j.joms.2008.04.022, 2-s2.0-49549103771. 18718396

